# Transcriptome Analysis Reveals Equine Endometrium’s Gene Expression Profile Around Embryo Fixation

**DOI:** 10.3390/genes16020181

**Published:** 2025-02-01

**Authors:** Tseweendolmaa Ulaangerel, Siqin Mu, Jolanqiqige Sodyelalt, Minna Yi, Bilig Zhao, Asiya Hao, Xin Wen, Baoxiang Han, Gerelchimeg Bou

**Affiliations:** 1 Equus Research Center, Inner Mongolia Agricultural University, Hohhot 010018, China; cewendclma@126.com (T.U.); siqin20220919@163.com (S.M.); julqg2022@163.com (J.S.); yiminna2020@163.com (M.Y.); bilig9@163.com (B.Z.); 15124926900@163.com (A.H.); wenxin618@imau.edu.cn (X.W.); 2 College of Veterinary Medicine, Inner Mongolia Agricultural University, Hohhot 010018, China; hanbaoxiang@imau.edu.cn; 3 College of Animal Science, Inner Mongolia Agricultural University, Hohhot 010018, China

**Keywords:** mare, embryo fixation, endometrium, transcriptome sequencing, gestation

## Abstract

Background/Objectives: The success or failure of embryo fixation is crucial for embryo attachment and later development. As an epithelial chorioallantoic placenta-type animal, the horse has a special process of embryo implantation, and the mechanism of embryo fixation in horses is still unclear. Methods: In this study, the structural and transcriptomic characteristics of endometrial tissue from the fixed and nonfixed sides of 20-day gestation embryos in Mongolian horses were investigated to search for important genes and potential molecular markers associated with the fixation phase of equine embryos. Results: A comparison of the structures of the endometrial tissues of the two sides revealed that the endometrium on the fixed side presented distinctive features, which were characterized mainly by the development of glands on the fixed side compared with those on the nonfixed side. A total of 3987 differentially expressed genes were identified in the transcriptome, among which 1931 genes were highly expressed on the fixed side of the embryo, including *CDH1*, *DRA*, *DQB*, *CLND2*, *BOLA-DQB*, *CLDN10*, *PTGER2*, and *PTGFR*. The differentially expressed genes were enriched in biological processes such as cell adhesion, morphogenesis, NOD signaling, and vitamin uptake, as well as prostatic hormones. Conclusions: These results suggest that equine embryo fixation may depend at least on the regulation of prostaglandins and the establishment of cellular connections. This provides a foundation for exploring the molecular mechanisms of key genes and pathways related to equine embryo fixation and offers new insights into feeding management and the monitoring of mares in the early stages of pregnancy.

## 1. Introduction

The structure and condition of the uterine lining play a vital role in the implantation of fertilized eggs and the healthy development of embryos. Once a fertilized egg is deposited, the mare uterus will support the development of the embryo through muscle contractions and changes in the lining. The embryo forms the placenta through its interaction with the uterine lining. The presence of a healthy, functional endometrium is critical throughout the mare’s gestation period, especially during the period of implantation and the long period prior to implantation, when the unimplanted fetus is completely dependent on exocrine secretion from the endometrial glands for life. Between days 6 and 16 after the fertilization of the ovum, the embryo migrates continuously through the uterine cavity to release maternal recognition signals across the entire surface of the endometrium. The embryonic movement stops abruptly on days 16–17, when the increasing diameter of the embryo prevents it from traveling further through the narrow uterine cavity [1]. It becomes “fixed” at the base of the uterine horns and is held firmly in place by increasing myometrial tension.

The cessation of embryonic activity is referred to as fixation and occurs at the caudal bend of one of the uterine horns. Fixation usually occurs abruptly, and the rate of embryonic activity or uterine contraction decreases as fixation approaches [1,2,3]. The degree of uterine contraction does not decrease significantly until the day of fixation, and the contraction continues to decrease after fixation until day 20 [4]. The choice of fixation angle may be a function of physical factors such as the embryo diameter, uterine tension, uterine diameter, and flexion of each caudal uterine horn. Fixation almost always occurs at the caudal flexure of the uterine horn because flexure is the greatest obstacle to embryo passage [1,5]. Embryonic fixation in horses and donkeys occurs at approximately day 16 of gestation [6]. In nonpregnant mares, fixation on the right side (~67%) is more common, which necessitates the determination of whether the right horn is smaller and more likely to preserve the embryo [7]. In lactating mares, the more frequent fixation on the left horn (~60%) is attributable, at least in part, to the fact that the horn of the previous pregnancy was the right horn of the mare; that is, the mare’s nonpregnant horn will be smaller and more complex after birth, meaning the embryo is more likely to fixate on the left side.

Embryo fixation is a prerequisite for successful embryo implantation and is regulated by various factors, such as cell adhesion molecules, tight junctions, and gap junctions. Embryo fixation plays a crucial role in pregnancy as a necessary preparatory event before equine implantation. However, there are almost no studies related to the mechanism of equine embryo fixation. We aimed to study the endometrium of the fixed and nonfixed sides of horses at 20 days of gestation (embryo fixation period) to understand the morphological differences, as well as the pattern of gene expression, and to identify pathways and genes that are highly expressed during this period to provide new ideas for the maintenance of pregnancy, feeding management, and the monitoring of female animals in the early stages of embryo gestation.

## 2. Materials and Methods

### 2.1. Collection of Mare Endometrial Tissue Samples

The experiments on animals were reviewed and approved by the Animal Protection and Utilization Committee of the Inner Mongolia Agricultural University. Endometrial tissues of three healthy Mongolian mares with 20 days of pregnancy were collected from the Dongwuzhumuqin Banner slaughterhouse in Xilingol League, Inner Mongolia. Samples of half of the endometrial tissues were snap-frozen in liquid nitrogen and stored at −80 °C until the analysis. The other half of the samples were fixed in paraformaldehyde and stored at −4 °C for the analysis.

### 2.2. The Histomorphologic of Endometrial Tissue in Mares

Mare endometrial tissues were fixed with 4% paraformaldehyde, dehydrated in graded ethanol, washed with xylene, paraffin-embedded, cut into 5-µm-thick sections, prepared, stained with hematoxylin–eosin, and photographed under a light microscope (Zeiss, Jena, Germany).

### 2.3. RNA Sequencing

The extraction of total RNA from tissue samples was performed using the TRIzol method, and the Nanodrop (Thermo Scientific, Waltham, MA, USA) and Agilent 2100 (Agilent, Santa Clara, CA, USA) instruments were used to test the purity and integrity of the total RNA. The Illumina RNA Library Prep Kit (NEB, Ipswich, MA, USA) was used to construct the libraries, and after the libraries were constructed, the quality, concentration, and size of the libraries were examined using Qubit 2.0 (Thermo Scientific, MA, USA) and Agilent 2100 to ensure the library’s quality. After meeting the expected standards, the libraries were sequenced via the Illumina HiSeqTM 2500 (Illumina, San Diego, CA, USA) platform.

### 2.4. Quality Control and Alignment

The sequencing data were filtered using SOAPnuke (v2.1.0) to ensure quality and confidence in the data analysis. After filtering, RNA-seq reads were mapped to the equine reference genome using HISAT2 (EquCab3.0). Second-generation sequence purification reads of controlled quality were compared to the reference transcript sequence using Bowtie2. The results of the Bowtie2 comparisons were tallied using RSEM (V1.3.1) to derive the number of reads per endometrial tissue sample compared to each transcript and converted to gene expression values (number of fragments per kilobase per million bases, FPKM), and the paired-end reads from the same fragment were counted as a single fragment to derive the gene and transcript expression levels.

### 2.5. Screening of DEGs

The FPKMs of all samples were subjected to a PCA to compare the FPKM density distributions of different sample genes and transcripts. Genes and transcripts with large differences in expression levels at different stages were selected via a statistical analysis of the expression data after a quantitative analysis.

### 2.6. Enrichment Analysis of GO and KEGG Pathways

The classification of genes and the subsequent annotation of their biofunctions was conducted using the online DAVID database. The DAVID database was then queried with symbols of differentially expressed genes between samples (adjusted *p* < 0.05). Gene Ontology (GO) annotation and Kyoto Encyclopedia of Genes and Genomes (KEGG) enrichment analyses were then used to analyze differentially expressed genes, as well as both upregulated and downregulated genes between samples. Equine genes that were tagged in the GO and KEGG databases were subsequently enriched and analyzed as background genes.

### 2.7. Quantitative Real-Time PCR (qRT-PCR) Assay

Total RNA from equine endometrial tissue was extracted using the Qiagen RNeasy Kit (Qiagen, Dusseldorf, Germany) and converted from RNA samples to cDNA using the High-Capacity cDNA Reverse Transcription Kit (Applied Biosystems, Waltham, MA, USA). The reaction mixture (20 µL) was prepared using TB Green^®^Premix Ex TaqTM II (Takara, Kyoto, Japan). *ACTB* was utilized as a control, and the reaction mixture (20 µL) was prepared using the TB Green^®^ Premix Ex TaqTM II instructions. The data were analyzed with the 2^−∆∆cT^ method to assess the levels of endometrial gene expression in Mongolian horses. Parallel experiments with at least three replicates were performed, and the data were expressed as the means ± SDs and analyzed using GraphPad Prism 9.0 software. Differences between groups were compared and were considered not significant at *p* < 0.05.

### 2.8. Immunohistochemistry

Dehydrated Mongolian horse endometrial samples (*n* = 6/group) were embedded in paraffin blocks. Using a microtome (RM 2245, Leica, Teaneck, NJ, USA), five-micron-thick cross-sections were taken from each block, placed on glass slides, and air-dried for 30 min. According to the protocol outlined in the antibody instruction manual, the antibodies were first diluted with antibody dilution buffer. Then, primary antibodies against *PTGFR* (Abcam, Waltham, MA, USA, 1:100), *PTGER2* (Affinity, West Bridgford, UK, 1:100), and *CDH1* (Affinity, 1:100) were incubated overnight at −4 °C. Following a rinse in phosphate-buffered saline (PBS) for 5 min, the slides were subjected to incubation with biotin-labeled secondary antibodies in a dropwise manner for 10 min at room temperature. Subsequently, a DAB horseradish peroxidase colorimetric kit (DA1015, Solarbio, Beijing, China) was utilized, and images were captured with a microscope imaging system (Zeiss, Germany).

### 2.9. Statistical Analysis

To evaluate significant differences in gene expression in the endometrial tissue in different states, the probability (*p*) values were evaluated through an analysis of variance (ANOVA). A difference was considered to be significant when *p* < 0.05.

## 3. Results

### 3.1. Comparison of Endometrial Histology

**Figure 1:**
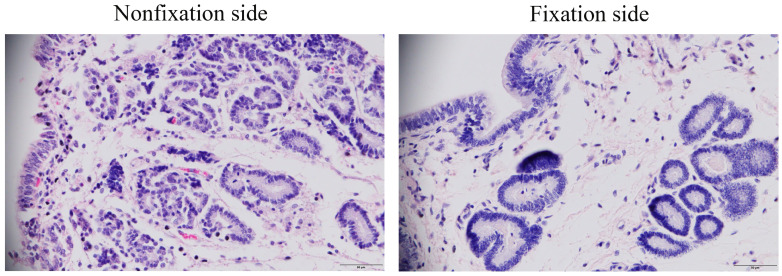
H&E staining of mare endometrial tissue on the fixed and nonfixed sides of the embryo. Scale bars, 50 µm.

We examined the morphology and microscopic features of the endometrium by performing H&E staining on paraffin sections of different tissue specimens. Morphological characteristics of the endometrium between the fixed side and the contralateral side of the embryo at 20 days of gestation are shown in Figure 1, where the endometrium of the fixed side was diffusely differentiated and the glands of the contralateral endometrium were hyperplastic differentiated. Compared with those on the contralateral side, the glands on the fixed side had larger openings, smaller gland diameters, more glandular ducts, enlarged glandular lumens, edematous endometrial stroma, and significantly lower surface gland density than did the glands in the endometrium on the nonfixed side. The morphological changes in the endometrium on the fixed side indicated that the endometrial secretory capacity was significantly enhanced via embryonic fixation.

### 3.2. Basic Statistical Analysis of the RNA Sequencing Data

To analyze the gene expression of the fixed side and nonfixed side of the embryo at 20 days of gestation, we sequenced 6 RNA libraries from the endometrial tissues of Mongolian horses at these two periods and performed quality control and filtering of the raw sequences on the sequencing platform. After removing contaminating and low-quality reads, we obtained 386,339,097 clean reads. Each sample’s clean base content exceeded 6,253,709,100 bp. More than 96.8% of the Q20 bases and more than 91.2% of the Q30 bases were enriched, while the filtered total GC content range was 49.6~51.0%, close to 50% (Table S1). The percentage of sequenced libraries compared with the reference genome was ≥96% for both the fixed and nonfixed sides of the embryos (Table S2).

### 3.3. Differential Expression Analysis

**Figure 2:**
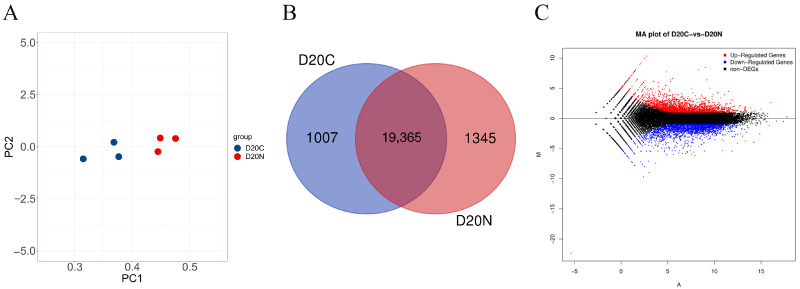
Quality assessment and basic statistics of the sequencing data: (**A**) a principal component analysis of endometrial tissue samples; (**B**) Wayne plot for the expressed genes in endometrial tissue samples from fixed and nonfixed sides of embryos; (**C**) volcano plot for the differentially expressed genes between the fixed and nonfixed sides of the embryo. D20C, fixed side of the embryo; D20N, nonfixed side of the embryo.

We performed a principal component analysis (PCA) on all samples, and the samples within the endometrial tissue groups of the fixed and nonfixed sides of 20-day-old embryos were clustered with each other, with a high degree of similarity in the expression patterns between samples and significant clustering with large intergroup differences (Figure 2A). The gene expression analysis revealed that 19,365 genes were common to the endometrial tissue of the fixed and nonfixed sides of the embryo at 20 days of gestation, 1007 genes were specific to the endometrial tissue of the fixed side of the embryo, and 1345 genes were specific to the endometrial tissue of the nonfixed side of the embryo (Figure 2B). In addition, 3987 genes were found to be expressed differentially between the fixed and nonfixed sides of equine embryos at 20 days of gestation. Of these, 1931 were highly expressed on the fixed side and 2056 on the nonfixed side at a threshold of *p* < 0.05 and |log2-fold change | ≥ 1 (Figure 2C).

### 3.4. Gene Function Enrichment Analysis

**Figure 3:**
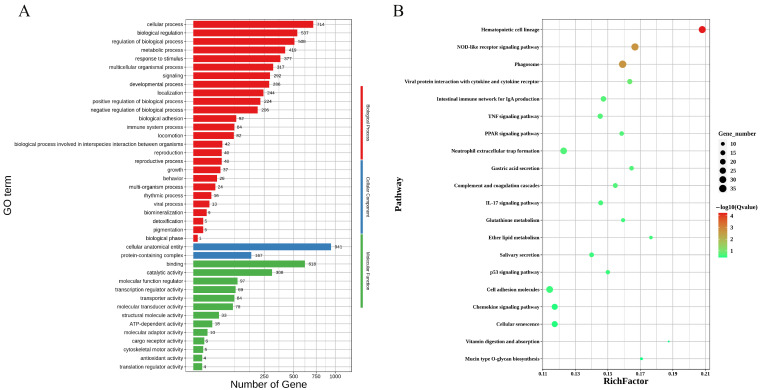
The enrichment analysis of highly expressed genes on the fixed side of the embryo: (**A**) the GO analysis of genes upregulated on the fixed side; (**B**) the KEGG pathway enrichment analysis of genes upregulated on the fixed side.

To gain a more comprehensive understanding of the identified DEGs, we performed a GO functional enrichment analysis using DAVID. A graphene oxide enrichment analysis of the highly expressed genes on the fixed side of the 20 day gestation embryos revealed that the upregulated genes were significantly enriched in 40 pathways, including 25 biological process pathways, 2 cellular constituent pathways, and 13 molecular function pathways. In the biological process (BP) category, the main processes include the cellular process, bioregulation, regulation of the biological process, the metabolic process, responses to stimuli, reproduction, the reproductive process, and adhesion; in the cellular component (CC) category, the main annotation is the protein-containing complex; and in the molecular function (MF) category, the main annotations include binding, catalytic activity, and cargo receptor activity. The GO annotations of the differentially expressed genes are shown in Figure 3A. To analyze the biological functions and metabolic pathways of the differentially expressed genes, the data were analyzed via KEGG pathway enrichment (*p* < 0.05). A total of 32 pathways were significantly enriched, including the NOD-like receptor signaling pathway, the TNF signaling pathway, the PPAR signaling pathway, the P53 signaling pathway, cell adhesion molecules, and other signaling pathways. The pathway annotations of some differentially expressed genes are shown in Figure 3B.

**Figure 4:**
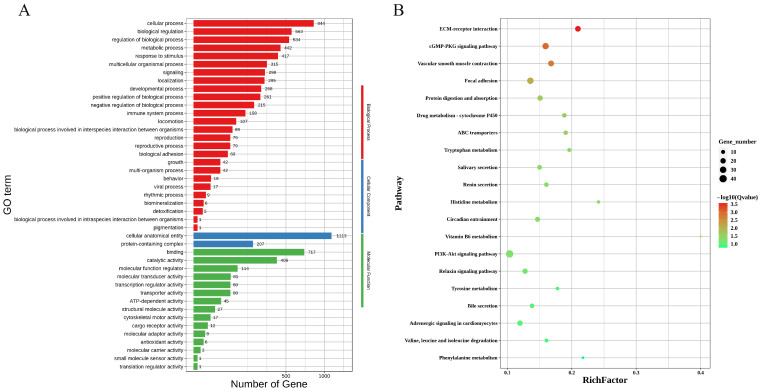
The enrichment analysis of highly expressed genes on the nonfixed side of the embryo: (**A**) the GO analysis of genes upregulated on the nonfixed side; (**B**) the KEGG pathway enrichment analysis of genes upregulated on the nonfixed side.

The GO enrichment analysis of highly expressed genes on the nonfixed side of 20 day gestation embryos from horses revealed that the upregulated genes were significantly enriched in 43 pathways, including 26 biological process pathways, 2 cellular component pathways, and 15 molecular function pathways. In the biological process (BP) category, the main processes include the cellular process, bioregulation, regulation of the biological process, the metabolic process, responses to stimuli, reproduction, the reproductive process, and adhesion; in the cellular component (CC) category, the main annotation is the protein-containing complex; and in the molecular function (MF) category, the main annotations include binding, catalytic activity, and cargo receptor activity. The GO annotations of the differential genes are shown in Figure 4A. The KEGG pathway enrichment analysis (*p* < 0.05) revealed that 32 pathways involving the cGMP-PKG signaling pathway, the PI3K-Akt signaling pathway, the relaxin signaling pathway, tyrosine metabolism, and other signaling pathways were significantly enriched. The pathway annotations of some differentially expressed genes are shown in Figure 4B.

### 3.5. Endometrium Gene Expression Analysis

**Figure 5:**
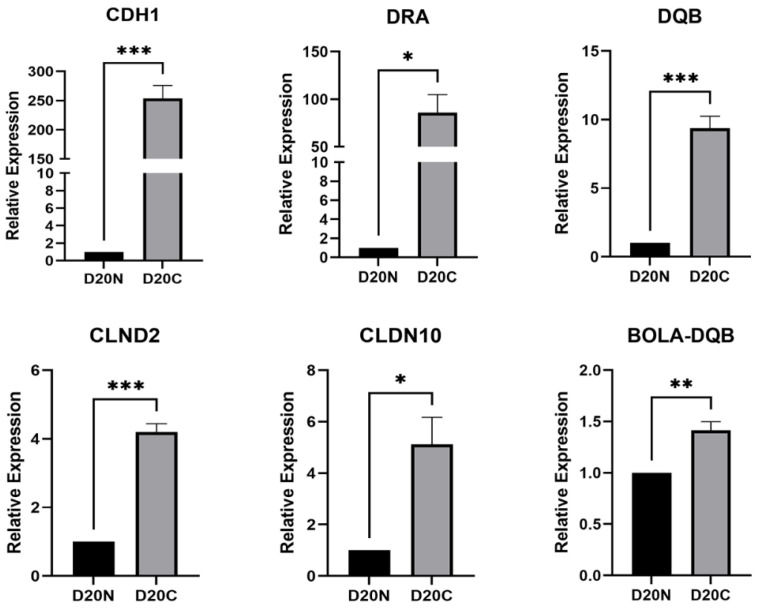
The qRT-PCR analysis of the *CDH1*, *DRA*, *DQB*, *CLND2*, *BOLA-DQB,* and *CLDN10* genes in the endometrial tissues of mares at 20 days of gestation; * indicates *p* < 0.05, ** indicates *p* < 0.01, *** indicates *p* < 0.001. The data are presented as the means ± SEMs (*n* = 3).

**Figure 6:**
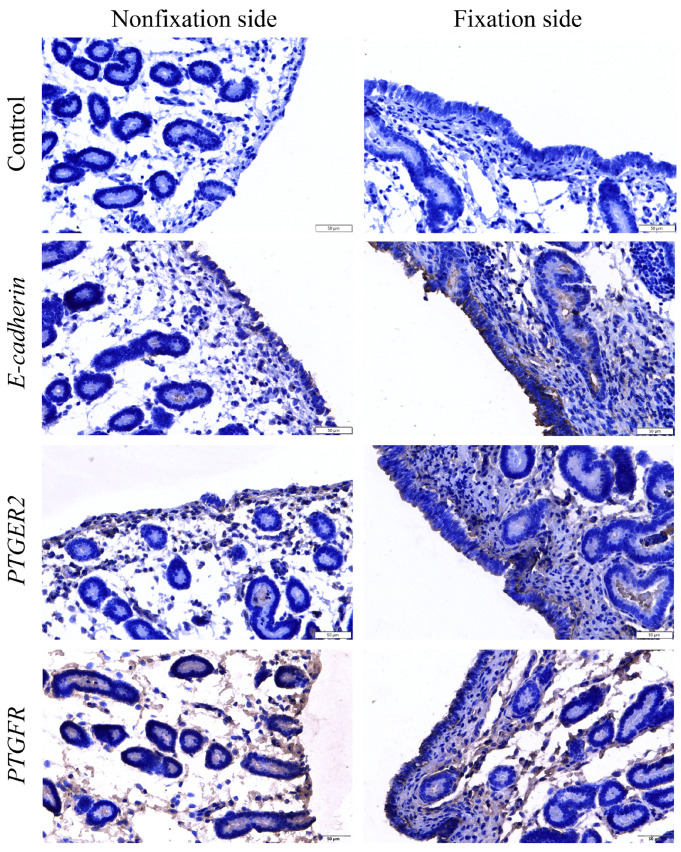
Immunohistochemical staining of E-cadherin (a *CDH-1* protein product), *PTGER2,* and *PTGFR* expression in endometrial tissues of mares at 20 days of gestation. Scale bars, 50 µm.

To study the gene expression in the endometrial tissues of the fixed and nonfixed sides of embryos at 20 days of gestation, we analyzed the genes that were differentially expressed between the two groups and selected 6 genes involved in the cell adhesion pathway, namely *CDH1*, *DRA*, *DQB*, *CLND2*, *BOLA-DQB*, and *CLDN10*, to perform qRT-PCR analyses to verify the accuracy of the transcriptome sequencing. Overall, the gene expression in endometrial tissues was consistent with the transcriptome sequencing results, and the mRNA expression of *CDH1*, *DRA*, *DQB*, *CLND2*, *BOLA-DQB*, and *CLDN10* was significantly greater on the fixed side than on the nonfixed side (Figure 5). In addition, the expression of E-cadherin (*CDH-1* protein product), *PTGER2*, and *PTGFR* in equine endometrial tissues was examined using an immunohistochemistry method, and the results were consistent with RNA sequencing, with E-cadherin and *PTGER*2 expressed at high levels on the fixed side of the embryo, whereas *PTGFR* was highly expressed in the endometrial tissues of the nonfixed side of the embryo (Figure 6). These results suggest that equine embryo fixation may be dependent on at least prostaglandin regulation and the establishment of cellular connections.

## 4. Discussion

Embryo implantation is a highly coordinated process among multiple tissue and cell types, such as embryonic trophoblast cells, the uterine epithelium, and endometrial stromal cells [8,9], and is generally believed to involve paracrine regulation mediated by the uterine fluid between multiple cells [10,11,12]. On the other hand, embryo fixation is a prerequisite for successful embryo implantation and is regulated by a variety of factors, including cell adhesion molecules, tight junctions, and gap junctions. Here, we sequenced the transcriptomes of endometrial tissues from the fixed and nonfixed sides of mare embryos at 20 days of gestation, with 3987 genes differentially expressed between the two tissues. Among these genes, 2056 were highly expressed on the nonfixed side of the embryo (Figure 2). The findings involved multiple GO classifications and the KEGG pathway. There were 1931 differentially expressed genes that were highly expressed on the fixed side of the embryo, including adhesion-associated *CDH1* and two receptor genes of the prostaglandin pathway, *PTGER2* and *PTGFR*.

E-cadherin (*CDH1*) is normally localized in the cytoplasm of the uterine glandular epithelium [13]. Studies on mice have shown that E-cadherin is also expressed in the embryo and that its absence results in enhanced embryonic swimming in the uterus [14,15,16]. E-cadherin expression in the uterine epithelium is involved in mechanical adhesion between epithelial cells and the maintenance of the normal epithelial structure [16], and it also plays an important role in endometrial tolerance. Additionally, in mice, E-cadherin was found to be expressed in focal contacts between peri-implantation embryos, uterine epithelial cells, trophoblast ectoderms, and the uterine epithelium at the time of implantation [17]. Studies have reported a significant increase in E-cadherin expression on the parietal membrane of mouse uterine epithelial cells before and at implantation [18], whereas knockout of the E-cadherin gene results in mouse embryos exhibiting preimplantation developmental defects and subsequent implantation failure [19]. The expression of E-cadherin by human embryonic trophoblast cells is associated with the establishment of a link between trophoblast cells and the endometrium, which is involved in the initial attachment phase of human embryo implantation [17,20,21,22,23]. In line with these findings, the endometrial expression of E-cadherin in women with recurrent miscarriage is reduced [13]. E-cadherin is clearly an important molecule that promotes embryo–maternal recognition and interactions during the early stages of embryo implantation. In our study, E-cadherin was significantly highly expressed on the endometrial tissue on the fixed side of the embryo at 20 days of gestation in horses, and this result is in line with related findings in other species, suggesting that E-cadherin has a conserved function in promoting embryo fixation on the endometrium in species with different bedding forms and placenta types.

Furthermore, our study revealed that prostaglandin E2 (*PGE2*) and prostaglandin F2*α* (*PGF2α*) may play important roles in equine embryo fixation. Equine embryos need to wander for a longer period of time prior to implantation, during which equine embryo-derived prostaglandins may be involved in maternal–fetal interactions to promote pregnancy-related events [24,25,26,27]. *PGE2* and *PGF2α* are involved in equine embryos throughout the uterus from day 1 to day 16 of gestation, and the movement of the embryo is driven by the embryo’s own secretion of *PGE2* and *PGF2α*, which stimulate uterine contractions that in turn drive the migration of the embryo within the endometrium. Moreover, *PGF2α* and *PGE2* originating from the endometrium also stimulate uterine contractions, which in turn drive the migration of the embryo within the endometrium. The release of endometrial *PGF2α* diminishes during maternal recognition of pregnancy (days 9 to 16 of gestation), after which the endometrium resumes its ability to secrete *PGF2α* during the third week of gestation. Prostaglandin secretion has been reported not only in horse embryos but also in embryos of other animals [26,27,28,29].

In most animals, when conception does not occur, luteolysis must be initiated to allow a new estrous cycle to begin. The function and integrity of the corpus luteum are necessary for the establishment and maintenance of pregnancy, so pregnancy must somehow inhibit luteolysis, a process known as maternal pregnancy recognition [30,31]. *PGF2α* is known to promote luteolysis. During a mare’s pregnancy, her *PGF2α* secretion decreases [32,33]. Consistent with these findings, the *PGF2α* levels in the uterine lumen of nonpregnant mares are much higher than those in the uterine lumen of mares at 14–16 days of gestation [34]. This finding is also in agreement with our finding that the level of the *PGF2α* receptor gene *PTGFR* in the endometrial tissue on the fixed side of the embryo was significantly lower in mares at 20 days of gestation than on the nonfixed side of the embryo. This may indicate that embryo–maternal exposure may also lead to reduced sensitivity by decreasing *PGF2α* receptor expression. In addition, *PGF2α* in semen is able to facilitate sperm transportation in the female reproductive tract by promoting uterine contractions [28]. In summary, the reduced expression of the *PGF2α* receptor gene *PTGFR* on the fixation side during the fixation period of equine embryos may favor the suppression of uterine contractions, thereby safeguarding embryo fixation.

*PGE2* functions in lysing the corpus luteum in the opposite way to *PGF2α*, which is thought to act in an anti-luteolysis manner. Therefore, we can infer that *PGE2* synthesis may play an important role in supporting pregnancy. The synthesis of *PGE2* by the maternal endometrium has been reported in many animals, including horses. In vitro studies have shown that embryos at days 1–15 can also produce *PGE2* and act on equine endometrial tissue [35]. Additionally, the uterus itself secretes *PGE2*, including the myometrium and endometrium [28,36,37]. Weber et al. [38,39] demonstrated that the ability of the oviduct to distinguish between developing embryos and unfertilized oocytes depends on the secretion of *PGE2* by the embryo from day 4 to day 5 after fertilization. Studies have shown that embryonic-derived *PGE2* may be one of the main drivers of uterine contractions [40]. Horse embryos produce large amounts of *PGE2* to stimulate uterine muscle contractions, which appear to help move the embryo to all regions of the uterus, allowing it to secrete an as yet unidentified anti-follicularulolytic signaling molecule [41].

In addition, very early embryos are unable to leave the fallopian tube because of the tense contraction of the isthmus circular smooth muscle, causing the junction of the juxtaglomerular isthmus to act as a closed sphincter. *PGE2* secreted by the developing mulberry embryo induces relaxation of the isthmus circular smooth muscle, leading to dilation of the isthmus sphincter and rapid passage of the embryo into the uterus [38,42]. These studies, in turn, suggest a role for *PGE2* in promoting muscle relaxation, which coincides with the significant increase in the expression of the *PGE2* receptor gene *PTGER2* on the fixation side of gestation on day 20 of pregnancy, suggesting that *PGE2* is likely to be an important endometrial and myometrial sedative during the embryo fixation phase in horses.

## 5. Conclusions

In conclusion, we investigated the expression differences between the transcriptomes of the endometrium on the fixed and nonfixed sides of the embryo using RNA sequencing technology. We identified 3987 DEGs, whose functional analyses are of theoretical and practical reference value for the in-depth study of the molecular mechanisms of key genes and pathways related to fixation in equine embryos.

## Data Availability

The original contributions presented in this study are included in the article/Supplementary Material. Further inquiries can be directed to the corresponding author.

## Supplementary Materials

The following supporting information can be downloaded at https://www.mdpi.com/article/10.3390/genes16020181/s1, Table S1. Quality control of mare endometrial tissue RNA-seq data. Table S2. Alignment of sequencing data with reference genomes. Table S3. Information of equine reference genome. Table S4. Primer information of qRT-PCR. Table S5. RNA sequencing data statistics.
