# Supplementary material for: Transcriptome Analysis Reveals Equine Endometrium’s Gene Expression Profile Around Embryo Fixation
Source: Genes (Basel). 2025 Feb 1;16(2):181. doi: 10.3390/genes16020181 (PMC11855126; doi:10.3390/genes16020181)
Supplement: Supplementary file 1 [file genes-16-00181-s001.zip › Supplementary Table S3.pdf]

Information of Equine reference genome

Genome: GCF_002863925.1_EquCab3.0_genomic.fna

https://www.ncbi.nlm.nih.gov/datasets/genome/GCF_002863925.1/
